# Ferulic acid and PDMS modified medical carbon materials for artificial joint prosthesis

**DOI:** 10.1371/journal.pone.0203542

**Published:** 2018-09-05

**Authors:** Xianlei Gao, Songgang Wang, Yeyang Xu, Hao Li, Hua Zhao, Xin Pan

**Affiliations:** 1 Department of Orthopaedics, Qilu Hospital of Shandong University, Jinan, China; 2 Cancer Research Center Shandong University, Jinan, China; Kyoto Daigaku, JAPAN

## Abstract

Medical carbon material has been extensively studied due to their excellent biological and mechanical properties. However, the dissociation of the surface carbon particles greatly limited the application of medical carbon material (MCM). To overcome this defect, we introduced the polydimethylsiloxane, a polymer-coating material (PCM) that possesses acceptable biocompatibility, into medical carbon material to prevent the shedding of carbon debris. Additionally, to reduce inflammatory reactions and increase surface hydrophilicity, ferulic acid, also called Chinese medicine coating material (CCM), was used to modify the surface of polymer-coating material. We investigated the proliferation and adhesion of NIH-3T3 cells onto MCM, PCM and CCM *in vitro*. We showed that CCM exhibited excellent biological activity to promote cell growth. Twelve weeks after CCM implantation, bone defects were repaired, and the material showed acceptable chemical stability. The results indicated that the CCM composite possesses excellent mechanical property and favorable biocompatibility, which can be used for clinical bone repair.

## Introduction

In recent years, the incidence of joint trauma caused by accidents or diseases has been increasing annually[1]. Owing to the limited self-repair capacity of bone tissue, joint injury will severely affect the quality of life of patients. Treatment of these injuries often requires surgery, which carries the risk of infections. With the improvement of medical technology, various composite materials are used in fabricating artificial joint prosthesis in orthopedic surgery, such as ceramic[2], bioactive glass[3] and metal[4], etc. However, the disadvantages of these materials as prostheses have limited the application of joint replacements with prosthesis made of the materials mentioned above.

Medical carbon material (MCM) is usually used as joint prosthesis biomaterials due to excellent biocompatibility and mechanical properties. It was reported that the main mechanical property of medical carbon material is more similar to that of natural bone than other common bone repair materials[5]. However, studies have shown that carbon particles produced by friction are often released from the interface between the implant and surrounding bone[6]. Although carbon debris can be phagocytized by macrophages and nearby lymph nodes[7] and are harmless to the human body, this phenomenon will reduce the mechanical strength and increase the inflammation risk. To circumvent this weakness, the bioactive layers are applied on the surface of the medical carbon materials, which can improve the mechanical strength of the implant and reinforce the integration with bone. Common bioactive coating and film materials include hydroxyapatite[8], tantalum coating[9], carbonado coating[10], and calcium phosphate (Cap)/collagen coatings[11]. Poly (dimethyl siloxane) (PDMS) is an ideal polymer coating that is impermeable to liquids. It has been widely used in the study of biocompatible surgical implants[12], microfluidic devices[13], and tissue engineering[14] due to the high transparency, biocompatibility, low fluorescence, and chemical inertness. Ferulic acid (FA) was found in the seeds and leaves of plants and is a ubiquitous natural phenolic phytochemical[15]. Ho Han *et al*. demonstrated the potential applicability of multiwall CNTs composited with PDMS, which could effectively promote neuronal differentiation and growth[16]. In addition, characterization of the PDMS/MWNT sheet showed superior mechanical strength and electrical conductivity to the bare PDMS.

Ferulic acid (FA) possesses various biological effects, such as anti-inflammatory[17], antibacterial[18], antioxidant[19], antiallergy[20], anti-cancer[21], and antiviral effects[22]. These biological functions mostly depend on the free radical scavenging and antioxidant activities of FA. The phenolic substances interact with a broad spectrum of target molecules in the cell signal transduction mechanism and play an important role in the regulation of cells. Additionally, previous research has shown that FA increased the viability of HEK293 cells and significantly reduces the apoptosis and oxidative damage induced by hydrogen peroxide[23].

In this study, we combined the advantages of medical carbon materials, polymer and Chinese medicine to investigate bone defect repair. Poly (dimethyl siloxane) was modified on the surface of medical carbon materials to form the polymer-coating material (PCM). Based on PCM, FA was further introduced on PCM to produce Chinese medicine coating material (CCM). Here, we report the mechanical properties, hydrophilicity and cell adhesion of MCM, PCM and CCM *in vitro*. Additionally, we studied the integration of the implant material with the surrounding bone tissue and formation of new bone *in vivo*.

## Materials and methods

### Materials and reagents

Medical carbon material was purchased from Jining Keneng carbon Mstar Technology Ltd (Jining, China), which is a type of carbon/carbon composite. Polydimethylsiloxane (PDMS) and the curing agent (Sylgard 184) were purchased from Dow Corning (U.S.). Ferulic acid was obtained from Jinan Cheng Sen Trading Co., Ltd (Jinan, China). Dulbecco’s modification of Dulbecco’s Eagle’s medium (DMEM) culture media was obtained from Thermo Fisher Biochemical Products Co., Ltd. (Beijing, China). Calcein AM was purchased from Sigma-Aldrich. Analytical reagents and double-distilled water were used to prepare the solutions. The NIH-3T3 cell line was purchased from Shanghai Ruijun Biological Technology Co. Ltd. (Shanghai, China). Rabbits were purchased from the Laboratory Animal Center of Shandong University (Jinan, China). Pentobarbital sodium and buprenorphine hydrochloride were purchased from Shanghai Longsheng Chemical co., ltd (Shanghai, China).

### Preparations of implant materials

Medical carbon material was immersed into the mixture of PDMS prepolymer and curing agent at a ratio of 9:1 for 8 h. The medical carbon material coated with PDMS was cured for 12 h in the oven to produce PCM. Next, PCM was incubated with FA solution (1 mg/mL) at room temperature for 4 h, producing CCM. After washing 3 times, extra FA on PCM was removed. Before the experiment, MCM, PCM and CCM were sterilized by ultraviolet light for 1 h.

### Material characterization

Scanning electron microscopy (SEM, JSM-6610LV, Japan Electronics Corporation) was used to scan the surface of the materials. The carbon/carbon composite embedded in PDMS (4-mm diameter and 10-mm depth) was examined by the Micro-CT (XWT-190-TC) system under the voltage of 100 kV. To detect the compressive strength of MCM and MCM modified with PDMS and FA, we used the microcomputer-controlled electronic universal testing machine (WDW-50E, China) in the experiment at a constant load rate. The wear-resistant properties were examined using an ultra-functional attrition-testing machine (UMT-2, Center for Tribology, Inc, USA). Eight samples were tested in each group, and the data was averaged. The contact angles of MCM, PCM and CCM were measured using the contact angle-measuring instrument (JY-PHb, China) at room temperature. Each sample was measured five times at different locations and then the data was averaged.

### Cell culture

The study was conducted to investigate whether MCM modified with PDMS and FA can promote the adhesion and proliferation of mammalian cells using the NIH-3T3 cell line, which is a mouse embryonic fibroblast cell line. NIH-3T3 cells were maintained in DMEM culture media supplemented with 10% fetal bovine serum, cultivated at 37°C and incubated under 5% CO_2_ conditions. Adherent cells were treated with trypsin. The cell suspension (200 μL) was added to the sterilized material (MCM, PCM and CCM) that was placed in a culture dish to investigate cell adhesion and proliferation. The samples were cultured for 24, 48 and 72 hours at a temperature of 37°C in 5% CO_2_. Additionally, adherent cells on the 3 materials were incubated with 1 μM calcein AM (excitation = 495 nm; emission = 515 nm) to stain live cells for 15 min at 37°C in 5% CO_2_. Cells were washed twice in PBS and were visualized under a laser scanning confocal microscope (LSM 780, Carl Zeiss AG, Germany). The cell fluorescence images were processed by ImageJ software.

To evaluate the biocompatibility of the three materials, cytotoxicity experiments *in vitro* were carried out based on the cell viability test. Sterilized samples were immersed in serum-free medium for 72 h at 37°C in an airtight container. The impregnation process was performed on a clean bench. The volume/surface area ratio of each sample was maintained at 3 cm^2^/mL. Industrial polyvinyl chloride (PVC) was used as a positive control. Following treatment with high-temperature steam, extracts were mixed with culture medium (10% fetal bovine serum and 90% DMEM) and were placed into sterile tubes. The cytotoxic effect of extracts from cell suspensions was estimated by the MTT test. The absorbance of each pore at 490 nm (OD) was determined by enzyme-linked immunosorbent assay at 4 h, 24 h, 48 h and 72 h. The cell viability formula was as follows: As: OD value of the experimental hole (cell and extract culture medium). Ac: OD value of the control hole (cell and culture medium). Ab: OD value of the blank hole (medium without cells and extract).

Cell survival rate=[(As−Ab)÷(Ac−Ab)]×100% (1)

*E*. *coli* (DH5 alpha strain) was used to study the bacteriostatic properties of the different samples. The prepared materials (MCM, PCM and CCM) were placed into the 24-well plates (five repeats/material). The 400-μL bacterium solutions were placed onto plates containing different materials in a sterile incubator at 37°C for 6 h. After incubation, the materials were gently rinsed with PBS twice. To test the activity of bacteria, the sample was dipped in 1.5 M PI solutions for 5 minutes. The bacteria on the materials were observed by confocal laser microscopy.

### Animals and ethics statement

**Fig 1:**
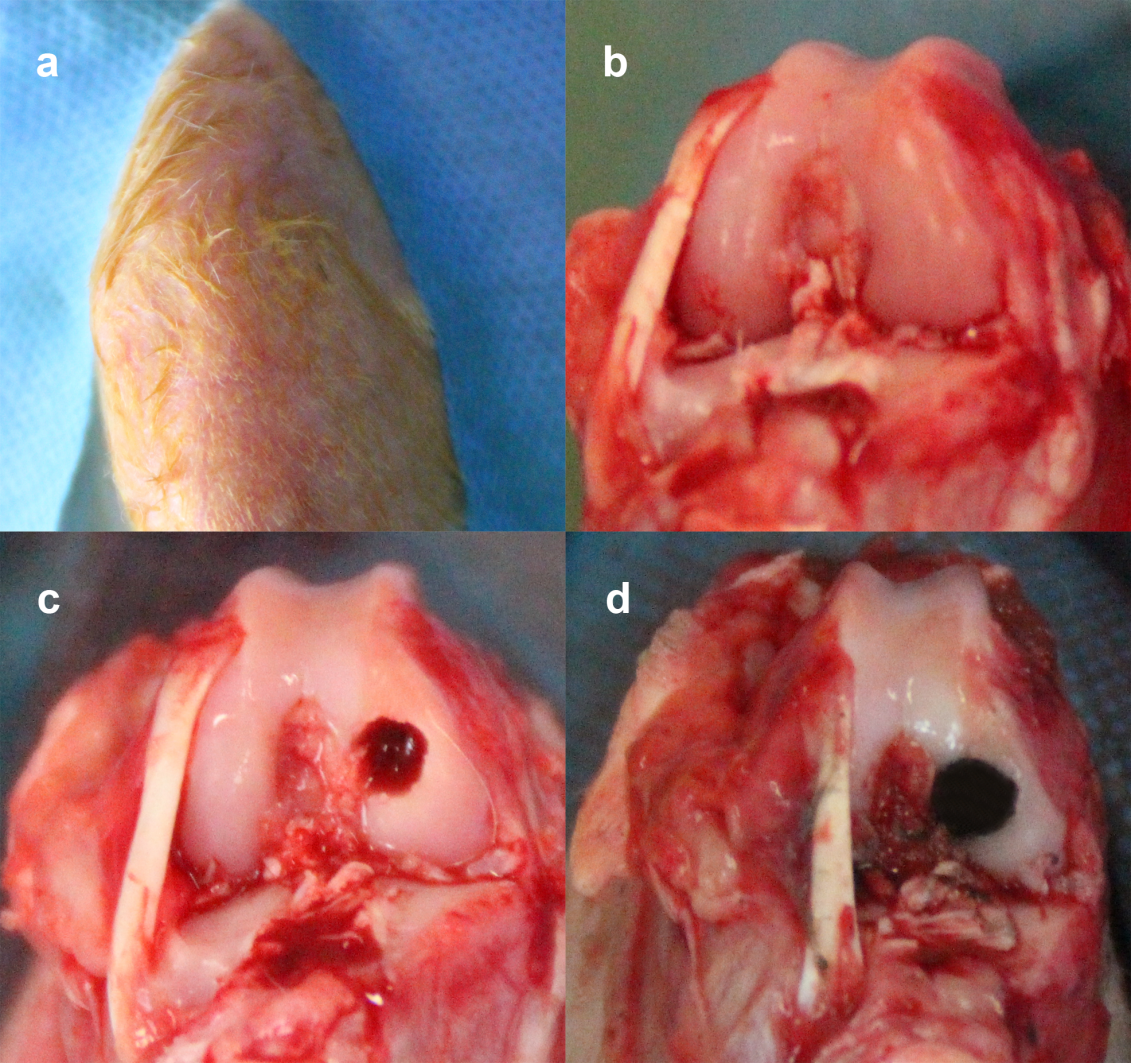
Implantation demonstration. (a) The surgery area is shaved and prepped in a sterile fashion, usually with the bated iodine solution. (b) The knees of animal were incised through a longitudinal medial patellar incision. (c) Subchondral drilling on the medial femoral condyle was performed on rabbits (4 mm diameter and 10 mm depth). (d) Implantation material was inserted into the defect with the articular surface.

New Zealand White rabbits weighing 2.5~3 kg were selected as an animal experimental model. The animal experiments were approved by the Animal Care Committee of Shandong University, following the Animal Management Rules of the Ministry of Health of the People’s Republic of China (document No 55, 2001). The rabbits were randomly divided into 4 groups, the sham group, MCM group, PCM group and CCM group, each with 3 rabbits. The rabbits were fasted routinely before undergoing surgery. Surgical tables and instruments must be disinfected. The animals were anesthetized under strict aseptic conditions through intramuscular injection of pentobarbital sodium (20 mg/kg). Under aseptic conditions, bone defects were constructed on the medial femoral condyle through drill holes (4 mm in diameter and 10 mm in height) (Fig 1). After surgery, the body temperature, heart rate and gastrointestinal motility were examined every two days. The animal is injected with buprenorphine hydrochloride (0.03 mg/kg) once a day for 3 days after surgery for analgesia. Surgical wound was checked to prevent wound infection daily. After 12 weeks, the rabbit administered euthanasia with an overdose of pentobarbital sodium (100 mg/kg, intraperitoneal injection).

### Histological evaluation

The rabbit joint containing composite material was placed in 15 mL centrifuge tubes with 10 mL 4% Paraformaldehyde and fixed for 24 hours on the decolorizing shaker at 4°C. After fixing, the rabbit joint was taken in 10% EDTA fixative solution for 90 days. After decalcification, the specimen dehydrates, and immerses the transparent examples in the wax. The samples were sliced in the sagittal plane with a 5-μm thickness. The frozen sections of joint samples were stained with hematoxylin and eosin (H&E) for histological studies and were observed under an optical microscope.

### Statistical analysis

All the data are expressed as the mean ± SD and were analyzed by GraphPad Prism software. One-way analysis of variance (ANOVA) was used to determine statistical significance. P values <0.05 were considered statistically significant.

## Results

### Material characterization

**Fig 2:**
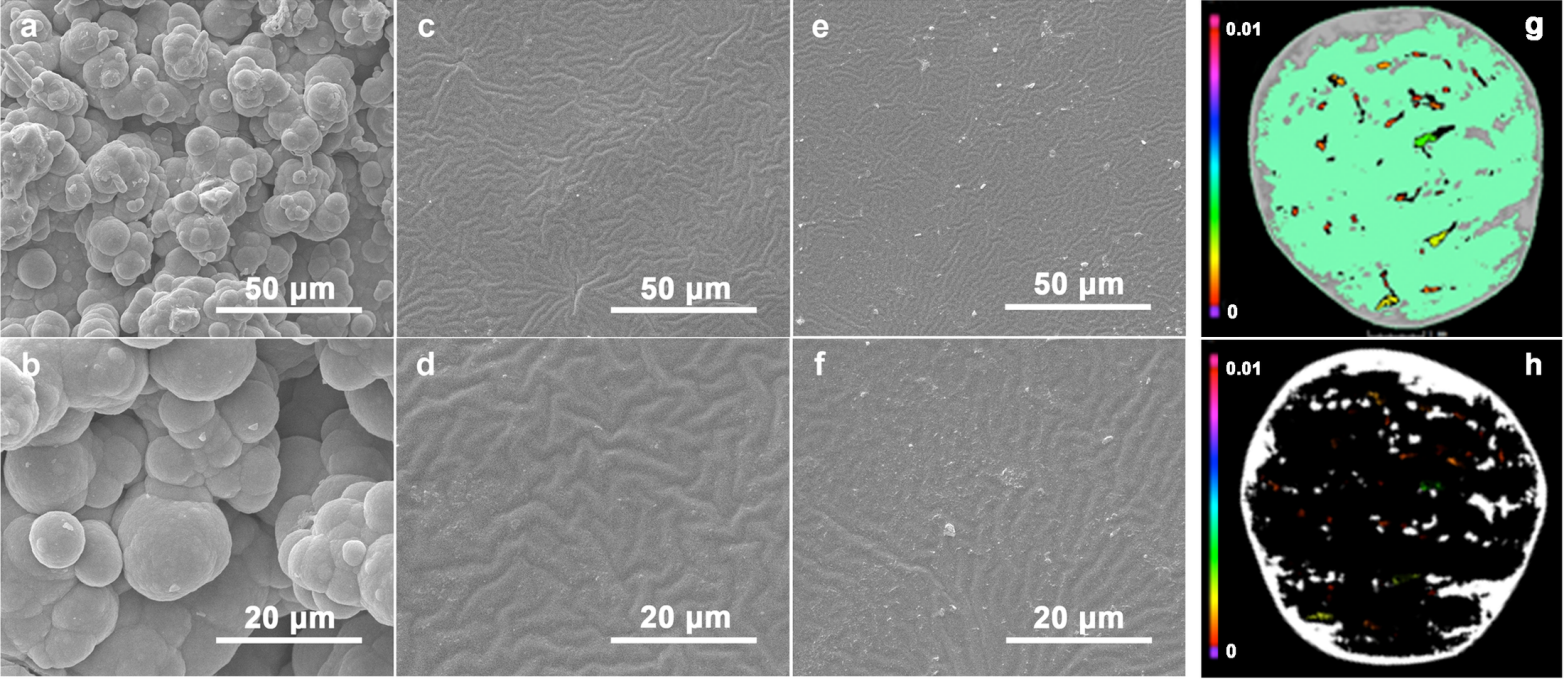
SEM images surface morphology of (a, b) MCM, (c, d) PCM and (e, f) CCM group. (b), (d) and (f) are the larger image of (a), (c) and (e), respectively. (g)&(h) Porosity distribution in the typical slice of PCM. C/C composite stands out in green, PDMS stands out in gray, porosities stands our in multicolor. Unit: mm^2^.

Scanning electron micrograph depicts the 3D surface topography of the three materials (Fig 2). The spherical surface rough particles were densely clustered and irregularly distributed on the surface of MCM (Fig 2). It is very challenging to obtain the smooth surface for implant materials due to the intrinsic defects of MCM. After soaking (PDMS polymer solution), the surface of MCM becomes smooth due to PDMS addition (Fig 2). Here, PDMS modified the surface to prevent ecclasis of carbon bits. To further increase the biocompatibility of the composite material, FA was modified onto the surface of PCM. However, the modification of FA stimulated no obvious change in the morphology as observed by SEM (Fig 2). From the micro-CT system image the degree of penetration of PDMS was revealed. The composite carbon material was prepared with PDMS, by soaking for a long time to be completely packed by PDMS. Additionally, some of the PDMS also penetrate the void of carbon materials.

### Hydrophilicity test

**Fig 3:**
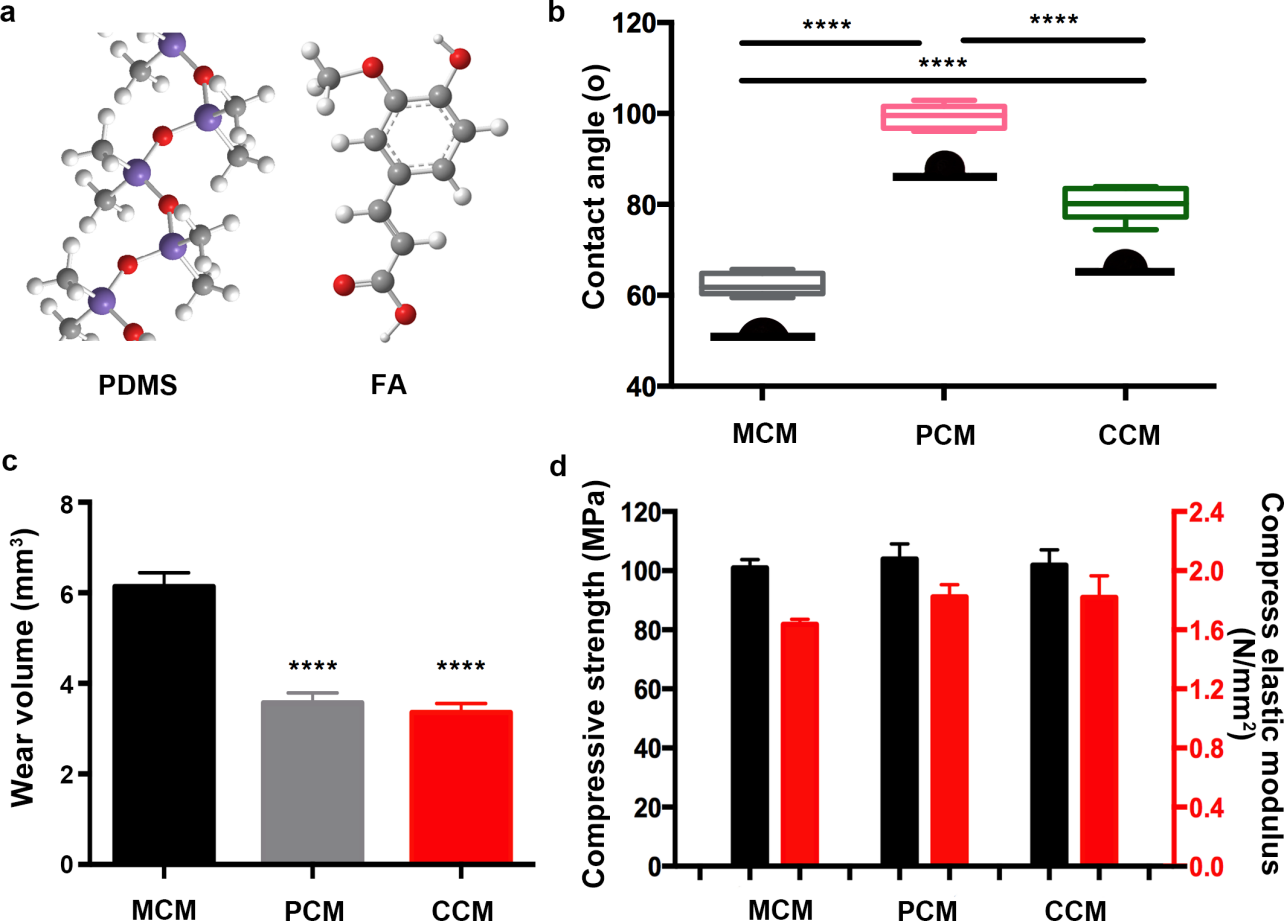
The physical characterization of MCM, PCM and CCM. (a) The chemical formula of PDMS and FA. (b) Material hydrophilicity test, (c) Wear volume and (d) Mechanical properties of MCM, PCM and CCM. ****: P<0.0001.

The contact angle of water is a convenient method to evaluate the hydrophobicity of the composite surface. Fig 3B shows the different changes in the contact angles of water drops to the MCM, PCM and CCM surfaces. The measurement of the contact angle has 10 replicates in each group to ensure the accuracy of the data. The contact angle of MCM with water is close to 62.4°(±2.4°). It also implies that the porous structure can increase the surface roughness and hydrophilicity. However, after the modification of PDMS, the contact angle of the PCM group with water increased. According to the results, PCM possesses the highest contact angle (99.4°±2.6°) corresponding to the lowest surface hydrophilicity. The addition of FA on the surfaces of PCM improves the biological properties of the materials, especially the hydrophilicity that signifies smaller contact angle of approximately 80.3° (±3.7°). The decrease in the contact angle on CCM may be related to the functional groups (Fig 3A) of FA[24].

### Mechanical properties measurement

Biomaterials used for bone repair usually have excellent mechanical properties to withstand the actual stresses in the body. The wear volume of MCM with a rough structure is lower than that of PCM and CCM with a smooth structure (Fig 3C). The difference was statistically significant. The increase in the wear volume may lead to the shedding of carbon bits, one of the causes of inflammation. The modification of PDMS on the surface of MCM can effectively prevent carbon dust shedding from MCM. The compressive strength of the three implants was 100.91±2.85, 103.93±5.11 and 101.84±5.25 MPa, respectively (Fig 3D). In statistical terms, the results indicate that there is no significant difference between each group. The data showed that the addition of PDMS and FA did not change the mechanical properties of the material.

### Bacteriostatic characteristics

**Fig 4:**
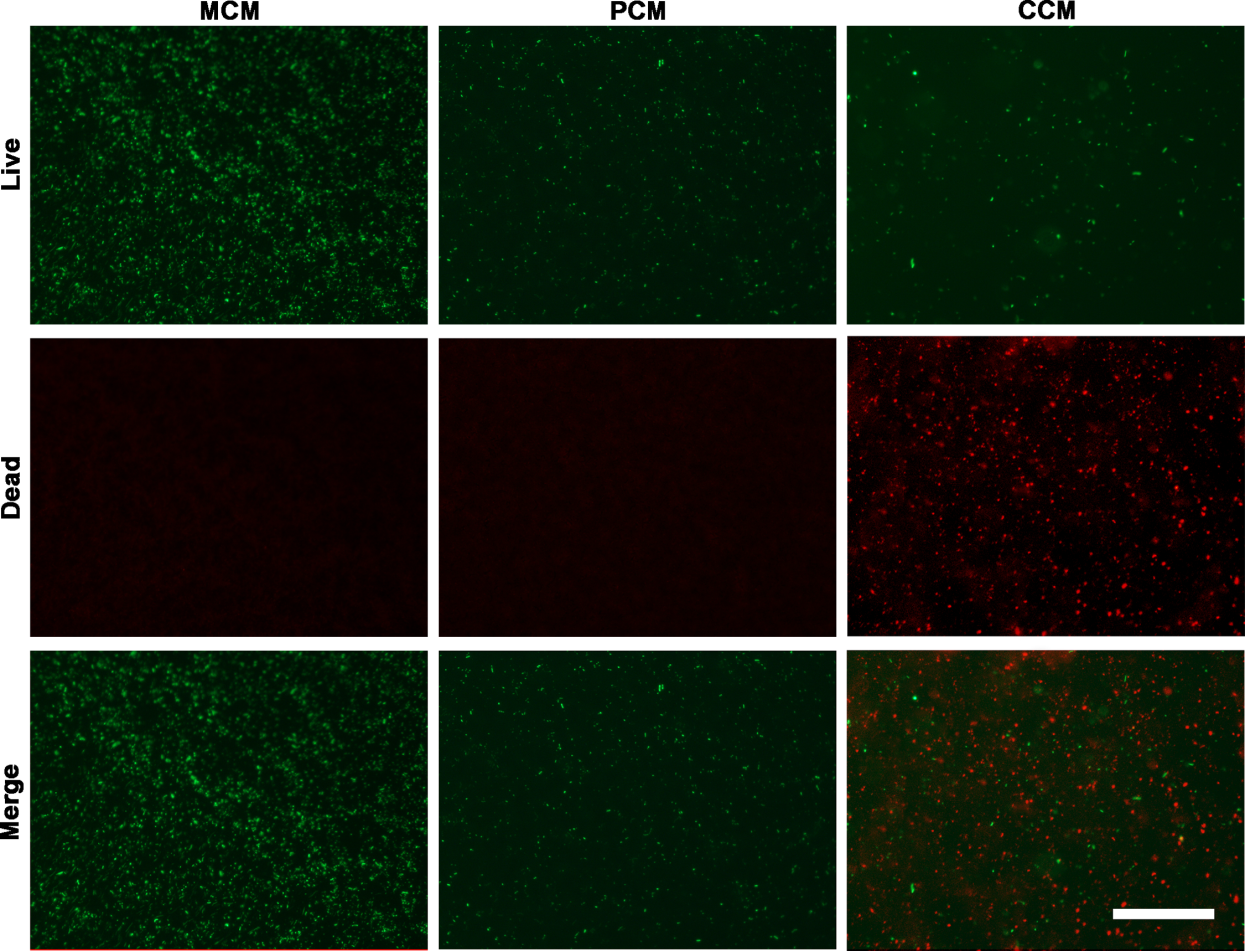
The antimicrobial properties on the surface of three substrates for 4 h. Scale bar: 100 μm. Dead bacteria are expressed in red, living bacteria expressed in green.

The antibacterial activity of MCM, PCM and CCM was evaluated against *E*. *coli* by comparing the inhibition of bacterial cells. *E*. *coli* DH5 alpha is a genetically modified bacterium that expresses green fluorescent protein and live green bacteria could be observed by fluorescent microscopy, while dead *E*. *coli* are dyed red by PI. In Fig 4, bacteria cells on MCM proliferated, indicating that this material has no anti-bacterial activity. Owing to the hydrophobicity of PDMS, the number of *E*. *coli* on PCM is less than that on other substrates. Compared with MCM and PCM, CCM effectively inhibited the growth of bacteria cells for 2 h where res fluorescence could be observed (S1 Fig). Bacteria death on CCM at 4 h was apparently higher than that on CMM at 2 h, indicating that the antimicrobial activity of FA persisted after 2 hours.

### Cell attachment test

**Fig 5:**
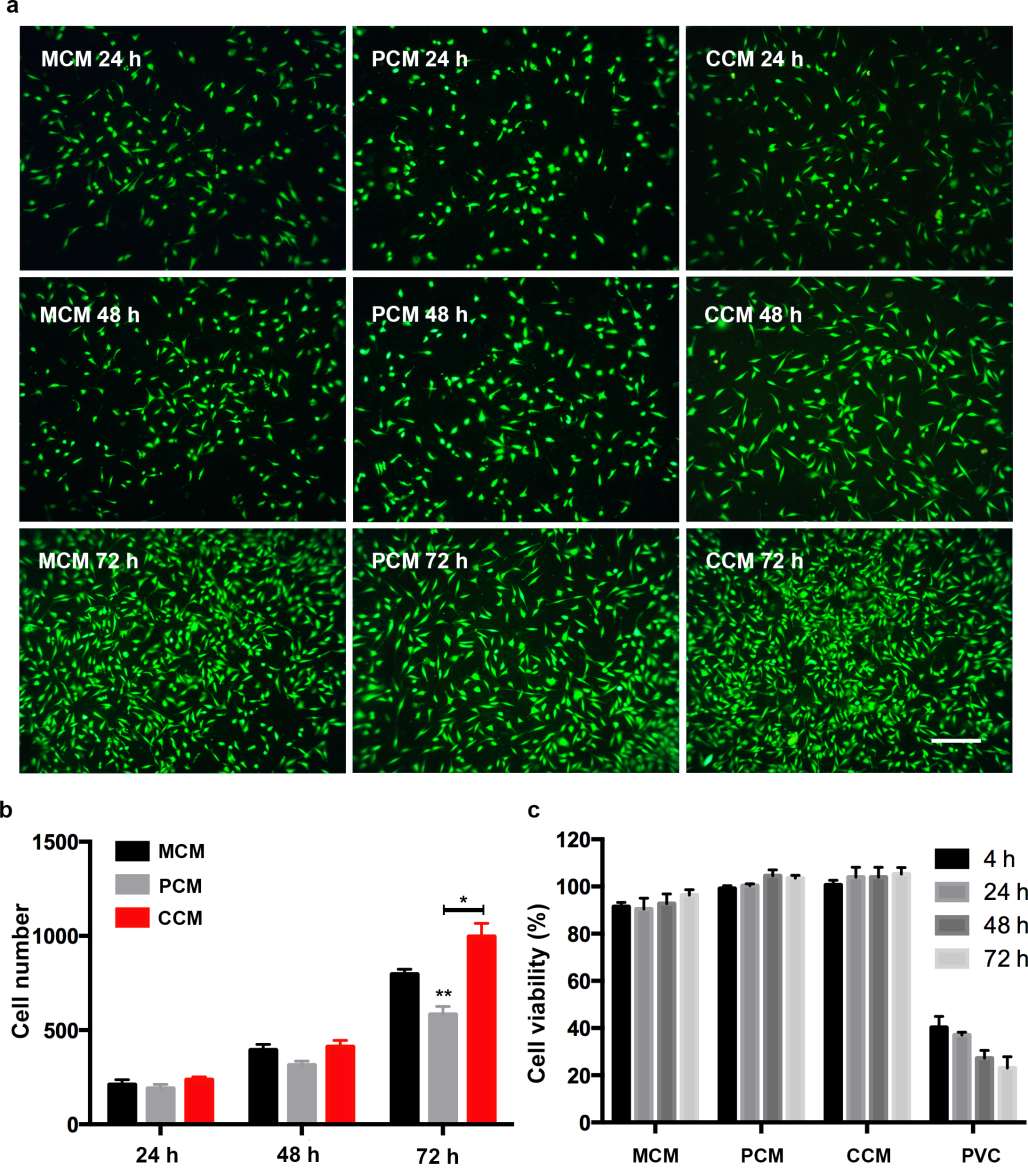
NIH-3T3 cell proliferation assay on MCM, PCM and CCM. (a) Fluorescence pictures that cells were cultured in three different matrix at 24, 48 and 72 h. (b) The number of NIH-3T3 cells adhered to the surface of materials at 24 h, 48 h and 72 h. (c) The cell viability of each test group (different leaching solution) at each observation time point. Scale bars: 100 μm. *: 0.01<P<0.005. **: 0.001<P<0.01.

Cell adhesion is the first stage of the interaction between cells and materials that can regulate cell proliferation, migration and differentiation. The growth of cells on the materials at 24 h, 48 h and 72 h is shown in Fig 5. As demonstrated in the diagram, the number of cells attached to various materials increased continuously with the increase in culture time (Fig 5B). At the same time, the cell number cultured on the CCM group was much higher than that on the MCM group and PCM group (Fig 5A). FA is a natural substance derived from plants and demonstrates an obvious cell proliferation effect[25]. Under the microscope, cells on CCM grew well with a typical spindle and polygon shape with a clear outline. The cells were in the stage of division and proliferation.

There was no significant difference in the number of cells cultured in three materials within 24 hours. As the incubation time increases, the number of cells among MCM, PCM and CCM has increased, especially at 72 h. MCM, PCM and CCM were tested by *in vitro* cytotoxicity as an orthopedic implant (Fig 5C). Three materials were soaked in culture medium to form the leachate extract. NIH-3T3 cells were exposed to extract for 4, 24, 48 and 72 h, and cytotoxicity was determined using the MTT assay. The results indicated that the cells with extracts of the three types of material exhibited good growth. The MTT assay showed that three kinds of material extracts did not have obvious cytotoxicity, especially CCM, at the same time incubation. The CCM composite demonstrates no cytotoxicity and good biocompatibility.

### Histological analysis

**Fig 6:**
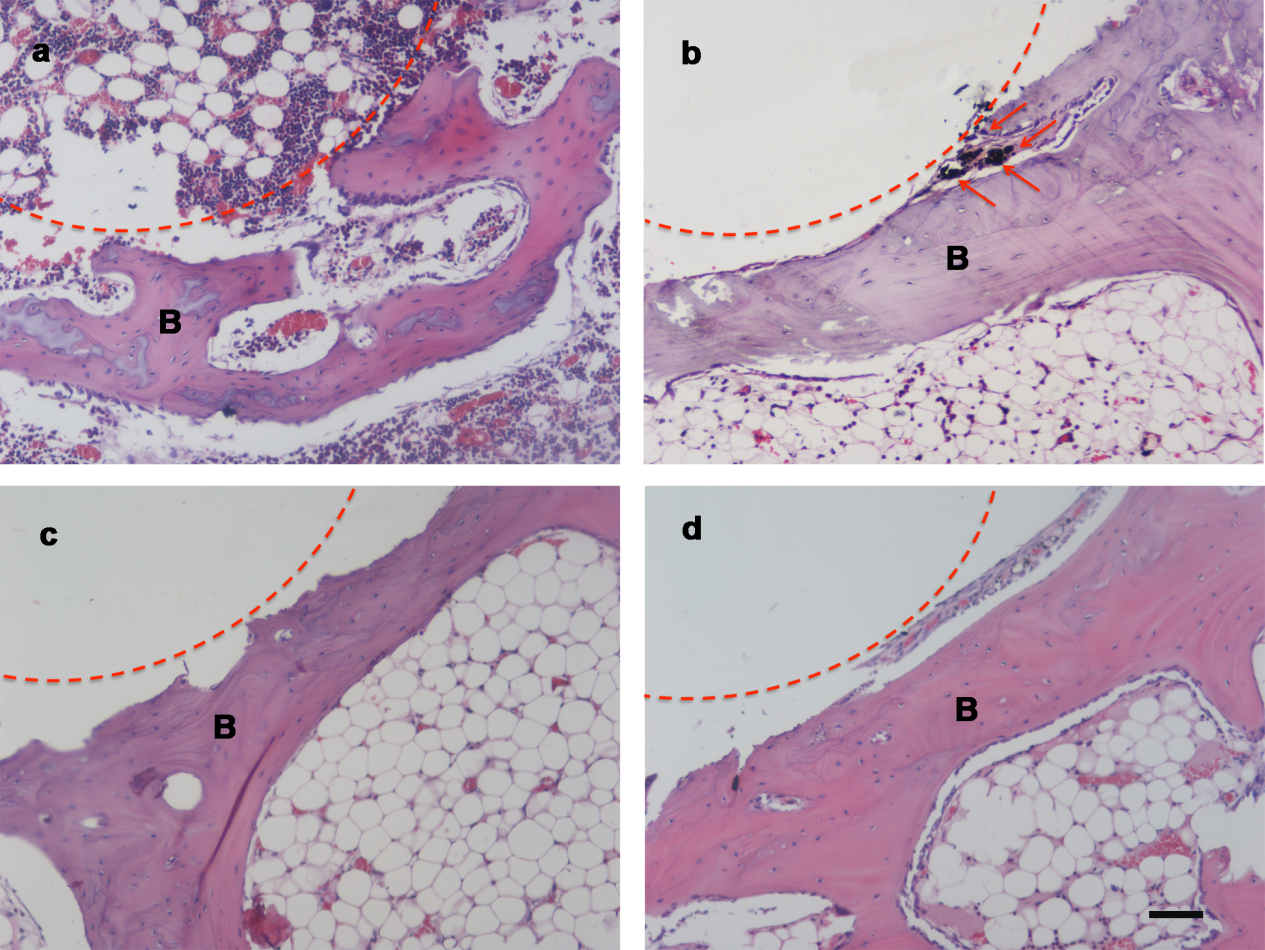
Histology of medial femoral condyle 12 weeks after implantation of blank, mcm, PCM and CCM (H&E staining), showing bone tissue growth. Scale bars: 100 μm. (a) The dotted line represents the punching site. (b-d) The dotted line represents the implant materials. B = bone.

To observe the *in vivo* condition of the implant, we performed histological analysis was performed. New bone tissue formation at the defect area was easily estimated by HE staining. As shown in the image, the bone rendered a deep red compact structure. There were more fibroblasts and scar tissue in the bone defect area in the control group under the light microscope (Fig 6A). The results showed that new bone formation was slow. Carbon filings are frequently found in MCM transplants experiments. As anticipated, it was obvious that carbon bits had shed from the carbon material and were distributed among bone tissue (Fig 6B). No obvious carbon particle abscission phenomenon was found in the PCM group by tissue section, as shown in Fig 6C. The results indicate that coating the surface of the carbon material could reduce carbon particle shedding on the carbon material surface. Additionally, we compared the condition of the new bone tissue around the implant group. In the CCM implant group, formation of new bone around the implant is more than that of the MCM and PCM group (Fig 6D). This proves that the addition of FA increases the possibility of new bone formation around the implant. The conclusion is consistent with the result of Fig 5 and further demonstrates the compatibility of CCM materials *in vivo*.

## Discussion

This paper presents the results of the physical property, biological compatibility and implantation experiments of MCM, PCM and CCM as artificial bone materials *in vivo* and *vitro*. The most important issue of using carbon materials as biomaterials is organizational compatibility and safety[26]. Bone sections results showed that there were a lot of carbon fragments in the bone tissue of MCM (Fig 6B). The carbon bits were equivalent to foreign materials in the body that easily induce a postoperative immune response and the risk of organism infections[27]. Many bioactive coatings and films have been prepared to increase the biological activity of carbon materials and prevent the release of carbon particles and broken carbon fibers[6], such as poly (-2hydroxyethyl methacrylate) film[28] and carborundum coating[29], etc. Because of the biocompatibility, PDMS has been extensively studied in surgical implants, microfluidic devices and tissue engineering[30]. However, due to the hydrophobicity of PDMS, the cells in the PCM group became shrunken and round in shape and gradually did not adhere well, likely causing the increase in the cell number decline on the PCM surface. The adhesion of cells to the material surface is important to mediate the biocompatibility of biomaterial, which could be influenced by the hydrophilic-hydrophobic property and surface morphology of material[31]. The presence of this antioxidant, FA, helps to increase the hydrophilicity of the membranes. Previous studies have shown that the contact angle of water droplets on the low-energy surface increases strongly with the increase of surface roughness and porosity[32]. PDMS increases the hydrophobicity of the carbon materials, but the modification of ferulic acid increases the hydrophilicity of the materials.

Plant chemical composition is the preferable candidate for antimicrobial therapy. Ferulic acid (FA) is a natural phenolic substance found in plant seeds, with a variety of biological activities, such as antioxidants, anti-inflammatory agents, antimicrobial agents, anti-allergy, liver-protecting agents, anticancer agents, antithrombotic agents[33]. It is reported that ferulic acid can affect the bacterial cell membrane, resulting in ion leakage and proton inflow, which may be the antibacterial mechanism of ferulic acid[34, 35]. Our results showed that the growth of bacteria was inhibited obviously after the addition of ferulic acid. Besides, ferulic acid could protect cells by inhibiting gene expression associated with cell death[36]. In our study, ferulic acid can effectively inhibit the growth of bacterial cells within 2 hours. In addition, the bacterial death of ferulic acid group at 4 hours was significantly higher than 2 hours, indicating that the ferulic acid 's antibacterial activity persisted after 2 hours.

There are many materials available as bone substitutes, such as natural and synthetic biodegradable polymers, ceramics, biological glass, metals and composites[37]. All of these materials have advantages and disadvantages. The natural, synthetic polymers and ceramics possess favorable biodegradability, biocompatibility, bioactivity and low mechanical strength[38–40]. Although metals have excellent mechanical properties (high strength and wear resistance, ductility), the lack of tissue adherence, corrosion and risk of toxicity due to release of metal ions limit the availability of the metals[41]. The main mechanical properties of this carbon material are closer to human bone than other common available bone repair materials[42]. The ideal coating material could improve the biological activity and mechanical property of MCM. Sung *et al*. found that PDMS can be an encapsulation material to develop a biocompatible, flexible and durable cable for prolonged implantation into the human body[43]. We compare the results of the wear volume and discuss the parameters of compressive strength and compress elastic moduli, as shown in Fig 3. Additionally, the compressed elastic modulus of PCM and CCM are greater than that of MCM, indicating that the PCM and CCM are not easy to deform compared with MCM. Therefore, PDMS can be used as a satisfactory material for the surface modification of MCM for *in vivo* implant. The results show that the PCM composites can effectively decrease the shedding of carbon particles and increase the wear resistance of the composites after the surface modification of PDMS on the MCM. In addition, the enhancing differentiation effect of the FA coating could accelerate the bone defect healing process and new bone formation. *In vivo* implantation and *in vitro* cell culture experiments showed that the CCM composite has good biocompatibility.

## Supporting information

S1 Fig The antimicrobial properties on the surface of three substrates for 2 h. Scale bar: 100 μm. Dead bacteria are expressed in red, living bacteria expressed in green.
(TIF)

## References

[1] Richmond SA, Fukuchi RK, Ezzat A, Schneider K, Schneider G, Emery CA. Are joint injury, sport activity, physical activity, obesity, or occupational activities predictors for osteoarthritis? A systematic review. Journal of Orthopaedic & Sports Physical Therapy. 2013;43(8):515. 23756344 10.2519/jospt.2013.4796

[2] Roy T, Choudhury D, Ghosh S, Mamat AB, Pingguan-Murphy B. Improved friction and wear performance of micro dimpled ceramic-on-ceramic interface for hip joint arthroplasty. Ceramics International. 2015;41(1):681–90.

[3] Chen Q, Baino F, Pugno NM, Vitalebrovarone C. Bonding strength of glass-ceramic trabecular-like coatings to ceramic substrates for prosthetic applications. Materials Science & Engineering C. 2013;33(3):1530–8. 23827605 10.1016/j.msec.2012.12.058

[4] Beckmann NA, Mueller S, Gondan M, Jaeger S, Reiner T, Bitsch RG. Treatment of severe bone defects during revision total knee arthroplasty with structural allografts and porous metal cones-a systematic review. Journal of Arthroplasty. 2015;30(2):249–53. 10.1016/j.arth.2014.09.016 25445853

[5] Sui JL, Li MS, Lü YP, Yin LW, Song YJ. Plasma-sprayed hydroxyapatite coatings on carbon/carbon composites. Surface & Coatings Technology. 2004;176(2):188–92.

[6] Cao N, Dong J, Wang Q, Ma Q, Xue C, Li M. An experimental bone defect healing with hydroxyapatite coating plasma sprayed on carbon/carbon composite implants. Surface & Coatings Technology. 2010;205(4):1150–6.

[7] Brandwood A, Noble KR, Schindhelm K. Phagocytosis of carbon particles by macrophages In vitro. Biomaterials. 1992;13(9):646–8. 1391413 10.1016/0142-9612(92)90035-m

[8] Cao N, Dong J, Wang Q, Ma Q, Wang F, Chen H, et al Plasma-sprayed hydroxyapatite coating on carbon/carbon composite scaffolds for bone tissue engineering and related tests in vivo. Journal of Biomedical Materials Research Part A. 2010;92A(3):1019–27. 10.1002/jbm.a.32424 19296542

[9] Li H, Zou X, Woo C, Ding M, Lind M, Bünger C. Experimental lumbar spine fusion with novel tantalum‐coated carbon fiber implant. Journal of Biomedical Materials Research Part B Applied Biomaterials. 2007;81B(1):194–200. 10.1002/jbm.b.30653 16924610

[10] Fu QG, Gu CG, Li HJ, Chu YH, Lu JH, Zhang LL. Microstructure and mechanical properties of SiC nanowires reinforced hydroxyapatite coating on carbon/carbon composites. Materials Science & Engineering A. 2013;563(7):133–7.

[11] Zhao X, Tao H, Li H, Chen M, Sheng C, Zhang L, et al Electrochemically assisted co-deposition of calcium phosphate/collagen coatings on carbon/carbon composites. Applied Surface Science. 2011;257(8):3612–9.

[12] Pérez-Tanoira R, García-Pedrazuela M, Hyyrynen T, Soininen A, Aarnisalo A, Nieminen MT, et al Effect of S53P4 bone substitute on staphylococcal adhesion and biofilm formation on other implant materials in normal and hypoxic conditions. Journal of Materials Science: Materials in Medicine. 2015;26(9):1–10. 10.1007/s10856-015-5569-1 26403279

[13] Sackmann EK, Fulton AL, Beebe DJ. The present and future role of microfluidics in biomedical research. Nature. 2014;507(7491):181–9. 10.1038/nature13118 24622198

[14] King KR, Terai H, Wang CC, Vacanti JP, Borenstein JT. Microfluidics for Tissue Engineering Microvasculature: Endothelial Cell Culture: Springer Netherlands; 2001 247–9 p.

[15] Kumar N, Pruthi V. Potential applications of ferulic acid from natural sources. Biotechnology Reports. 2014;4(1):86–93. 28626667 10.1016/j.btre.2014.09.002 PMC5466124

[16] Han IH, Sun F, Choi YJ, Zou F, Nam KH, Cho WH, et al Cultures of Schwann-like cells differentiated from adipose-derived stem cells on PDMS/MWNT sheets as a scaffold for peripheral nerve regeneration. Journal of Biomedical Materials Research Part A. 2015;103(11):3642 10.1002/jbm.a.35488 25903927

[17] Nile SH, Ko EY, Kim DH, Keum YS. Screening of ferulic acid related compounds as inhibitors of xanthine oxidase and cyclooxygenase-2 with anti-inflammatory activity. Revista Brasileira De Farmacognosia. 2016;26(1):50–5.

[18] Borges A, Ferreira C, Saavedra MJ, Simões M. Antibacterial activity and mode of action of ferulic and gallic acids against pathogenic bacteria. Microbial Drug Resistance. 2013;19(4):256–65. 10.1089/mdr.2012.0244 23480526

[19] Aceituno-Medina M, Mendoza S, Rodríguez BA, Lagaron JM, López-Rubio A. Improved antioxidant capacity of quercetin and ferulic acid during in-vitro digestion through encapsulation within food-grade electrospun fibers. Journal of Functional Foods. 2015;12:332–41.

[20] Lee CC, Wang CC, Huang HM, Lin CL, Leu SJ, Lee YL. Ferulic Acid Induces Th1 Responses by Modulating the Function of Dendritic Cells and Ameliorates Th2-Mediated Allergic Airway Inflammation in Mice. Evidence-based Complementary and Alternative Medicine. 2015;2015(4):678487. 26495021 10.1155/2015/678487 PMC4606409

[21] Cai Y, Luo Q, Sun M, Corke H. Antioxidant activity and phenolic compounds of 112 traditional Chinese medicinal plants associated with anticancer. Life Sciences. 2004;74(17):2157–84. 10.1016/j.lfs.2003.09.047 14969719 PMC7126989

[22] Spasova M, Philipov S, Nikolaeva-Glomb L, Galabov AS, Ts M. Cinnamoyl- and hydroxycinnamoyl amides of glaucine and their antioxidative and antiviral activities. Bioorg Med Chem. 2008;16(15):7457–61. 10.1016/j.bmc.2008.06.010 18590964

[23] Bian YY, Guo J, Majeed H, Zhu KX, Guo XN, Peng W, et al Ferulic acid renders protection to HEK293 cells against oxidative damage and apoptosis induced by hydrogen peroxide. In Vitro Cellular & Developmental Biology—Animal. 2015;51(7):722. 25678463 10.1007/s11626-015-9876-0

[24] Wang L, He S, Wu X, Liang S, Mu Z, Wei J, et al Polyetheretherketone/nano-fluorohydroxyapatite composite with antimicrobial activity and osseointegration properties. 2014;35(25):6758–75. 10.1016/j.biomaterials.2014.04.085 24835045

[25] Yabe T, Hharada H. Ferulic acid induces neural progenitor cell proliferation in vitro and in vivo. Neuroscience. 2010;165(2):515–24. 10.1016/j.neuroscience.2009.10.023 19837139

[26] Usui Y, Aoki K, Narita N, Murakami N, Nakamura I, Nakamura K, et al Carbon nanotubes with high bone-tissue compatibility and bone-formation acceleration effects. Small. 2008;4(2):240–6. 10.1002/smll.200700670 18205152

[27] Wang QS, Cui YL, Gao LN, Guo Y, Li RX, Zhang XZ. Reduction of the pro‐inflammatory response by tetrandrine‐loading poly(l‐lactic acid) films in vitro and in vivo. Journal of Biomedical Materials Research Part A. 2014;102(11):4098–107. 10.1002/jbm.a.35083 24442958

[28] Pesáková V, Jr SK, Sochor M, Hulejová H, Balík K. Biological properties of the intervertebral cages made of titanium containing a carbon-carbon composite covered with different polymers. Journal of Materials Science Materials in Medicine. 2005;16(2):143–8. 10.1007/s10856-005-5933-7 15744602

[29] Fu QG, Li HJ, Shi XH, Li KZ, Wang C, Huang M. Double-layer oxidation protective SiC/glass coatings for carbon/carbon composites. Surface & Coatings Technology. 2006;200(11):3473–7.

[30] Peterson SL, McDonald A, Gourley PL, Sasaki DY. Poly(dimethylsiloxane) thin films as biocompatible coatings for microfluidic devices: cell culture and flow studies with glial cells. Journal of Biomedical Materials Research Part A. 2005;72A(1):10–8. 10.1002/jbm.a.30166 15534867

[31] Pesáková V, Klézl Z, Balík K, Adam M. Biomechanical and biological properties of the implant material carbon-carbon composite covered with pyrolytic carbon. Journal of Materials Science: Materials in Medicine. 2000;11(12):793–8. 15348062 10.1023/a:1008953529111

[32] Hsieh CT, Chen JM, Kuo RR, Lin TS, Wu CF. Influence of surface roughness on water- and oil-repellent surfaces coated with nanoparticles. Applied Surface Science. 2005;240(1):318–26.

[33] Kumar N, Pruthi V. Potential applications of ferulic acid from natural sources. Biotechnology Reports. 2014;4:86–93. 10.1016/j.btre.2014.09.002. 28626667 PMC5466124

[34] Shi C, Zhang X, Sun Y, Yang M, Song K, Zheng Z, et al Antimicrobial Activity of Ferulic Acid Against Cronobacter sakazakii and Possible Mechanism of Action. Foodborne Pathogens and Disease. 2016;13(4):196–204. 10.1089/fpd.2015.1992 . 26919471

[35] Anabela B, Carla F, Maria JS, Manuel S. Antibacterial Activity and Mode of Action of Ferulic and Gallic Acids Against Pathogenic Bacteria. Microbial Drug Resistance. 2013;19(4):256–65. 10.1089/mdr.2012.0244 . 23480526

[36] Song Y, Wen L, Sun J, Bai W, Jiao R, Hu Y, et al Cytoprotective mechanism of ferulic acid against high glucose-induced oxidative stress in cardiomyocytes and hepatocytes. Food & Nutrition Research. 2016;60:30323. 26869273 10.3402/fnr.v60.30323 PMC4751457

[37] Samavedi S, Whittington AR, Goldstein AS. Calcium phosphate ceramics in bone tissue engineering: A review of properties and their influence on cell behavior. Acta Biomaterialia. 2013;9(9):8037 10.1016/j.actbio.2013.06.014 23791671

[38] Nampoothiri KM, Nair NR, John RP. An overview of the recent developments in polylactide (PLA) research. Bioresource Technology. 2010;101(22):8493–501. 10.1016/j.biortech.2010.05.092 20630747

[39] Lanao RPF, Jonker AM, Wolke JGC, Jansen JA, Hest JCMV, Leeuwenburgh. Physicochemical Properties and Applications of Poly(lactic-co-glycolic acid) for Use in Bone Regeneration. Tissue Engineering Part B Reviews. 2013;19(4):380–90. 10.1089/ten.TEB.2012.0443 23350707 PMC3690090

[40] Wang J, Chen Y, Zhu X, Yuan T, Tan Y, Fan Y, et al Effect of phase composition on protein adsorption and osteoinduction of porous calcium phosphate ceramics in mice. Journal of Biomedical Materials Research Part A. 2014;102(12):4234 10.1002/jbm.a.35102 24497384

[41] García-Gareta E, Coathup MJ, Blunn GW. Osteoinduction of bone grafting materials for bone repair and regeneration. Bone. 2015;81:112 10.1016/j.bone.2015.07.007 26163110

[42] Li S, Zheng Z, Liu Q, de Wijn JR, De GK. Collagen/apatite coating on 3-dimensional carbon/carbon composite. Journal of Biomedical Materials Research Part A. 2015;40(4):520–9. 10.1002/(sici)1097-4636(19980615)40:4<520::aid-jbm2>3.0.co;2-h 9599027

[43] Kim SH, Moon JH, Kim JH, Jeong SM, Lee SH. Flexible, stretchable and implantable PDMS encapsulated cable for implantable medical device. Biomedical Engineering Letters. 2011;1(3):199–203.

