# Supplementary figures and images for: Ferulic acid and PDMS modified medical carbon materials for artificial joint prosthesis
Source: PLoS One. 2018 Sep 5;13(9):e0203542. doi: 10.1371/journal.pone.0203542 (PMC6124784; doi:10.1371/journal.pone.0203542)

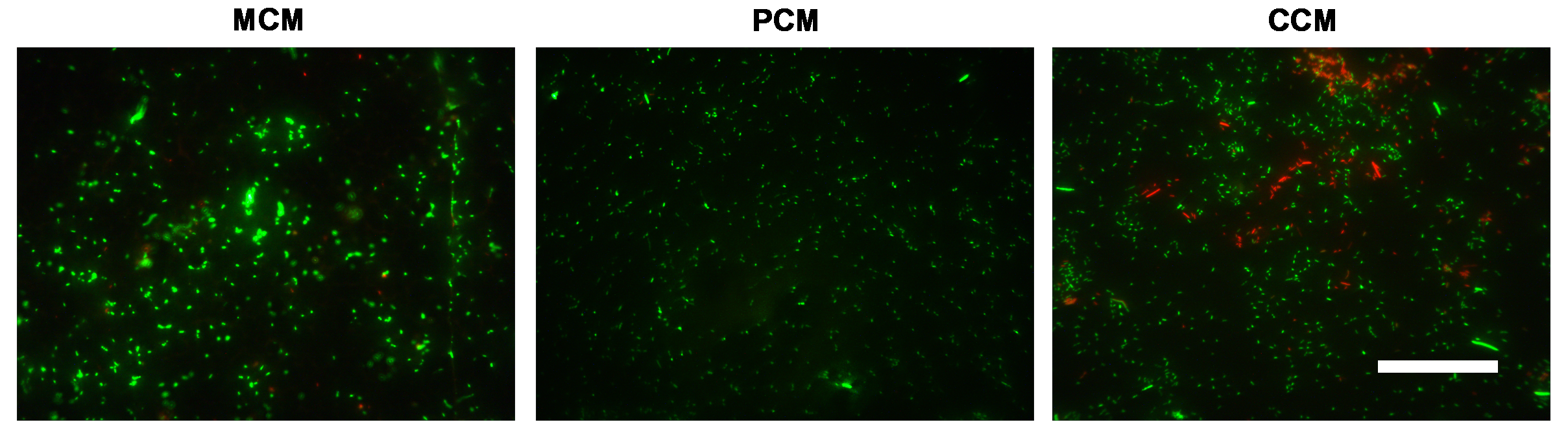

Supplement: S1 Fig — Scale bar: 100 μm. Dead bacteria are expressed in red, living bacteria expressed in green. (TIF) [file pone.0203542.s001.tif]
